# Everolimus and sunitinib for advanced pancreatic neuroendocrine tumors: a matching-adjusted indirect comparison

**DOI:** 10.1186/2162-3619-2-32

**Published:** 2013-12-06

**Authors:** James Signorovitch, Elyse Swallow, Evan Kantor, Xufang Wang, Judith Klimovsky, Tomas Haas, Beth Devine, Peter Metrakos

**Affiliations:** 1 Analysis Group, Inc., 111 Huntington Ave 10th Floor, Boston, MA 02199, USA; 2 Novartis Pharmaceuticals Corporation, 180 Park Ave, Bldg 105, Florham Park, NJ 07932, USA; 3 Novartis Oncology, Postfach, CH-4002, Basel, Switzerland; 4 School of Pharmacy, University of Washington, Box 357630, 1959 NE Pacific St, Seattle, WA 98195, USA; 5 McGill University Health Center, Multi-Organ Transplant and Hepatopancreatobiliary Service, Royal Victoria Hospital, 687 Pine Ave West, Montréal, (Québec) H3A 1A1, Canada

**Keywords:** Pancreatic neuroendocrine tumors, Everolimus, Sunitinib, Indirect comparison, Cross-over

## Abstract

**Background:**

Everolimus and sunitinib have been approved for the treatment advanced pancreatic neuroendocrine tumors, but have not been compared to each other in a randomized trial and have not demonstrated prolonged overall survival compared to placebo. This study aimed to indirectly compare overall and progression-free among everolimus, sunitinib and placebo across separate randomized trials.

**Methods:**

A matching adjusted indirect comparison was conducted in which individual patient data from the pivotal trial of everolimus (n = 410) were adjusted to match the inclusion criteria and average baseline characteristics reported for the pivotal trial of sunitinib (n = 171). Prior to matching, trial populations differed in baseline performance status and prior treatments. After matching, these and all other available baseline characteristics were balanced between trials.

**Results:**

Compared to the placebo arm in the sunitinib trial, everolimus was associated with significantly prolonged overall survival (HR = 0.61, 95% CI = 0.38-0.98, p = 0.042).

Compared to sunitinib, everolimus was associated with similar progression-free (hazard ratio for death (HR) = 0.84, 95% CI = 0.46–1.53, p = 0.578) and overall survival (HR = 0.81, 95% CI = 0.49–1.31, p = 0.383).

**Conclusion:**

After adjusting for observed cross-trial differences, everolimus treatment was associated with longer overall survival than the placebo arm in the sunitinib trial for advanced pancreatic neuroendocrine tumors.

## Background

Pancreatic neuroendocrine tumors (pNETs) are diagnosed in one of every 300,000 people per year. In the majority of presentations, the tumor is metastatic and unresectable [1]. Until recently, these patients faced limited treatment options and typically a median survival of less than 2 years [1-4]. In 2011, two biologically-targeted therapies, everolimus and sunitinib, were approved for the treatment of advanced pNET [5-10] and recommended for patients with progressive tumors that are locoregional, unresectable and/or metastatic [11]. Both treatments were shown to prolong progression-free survival in clinical trials. However, as is often the case for new treatments in oncology, randomized trials have not compared these treatments to each other and have not established effects on overall survival (OS). In addition, sunitinib was transiently associated with prolonged overall survival compared to placebo, in a trial stopped early following an unplanned data look [12-14], raising the question of whether there is stronger evidence for an OS benefit for sunitinib than for everolimus in advanced pNET. These evidence gaps can complicate decision making in advanced pNET for patients, physicians and payers.

In the absence of a direct head-to-head trial, outcomes can be compared across separate trials. Formal methods for such indirect comparisons, and extensions to network meta-analyses of multiple treatments, have been used increasingly, and guidelines for use have been developed [15,16]. However, it is well-recognized that cross-trial differences in patient populations or study designs can bias indirect comparisons [15-19]. This limitation is especially pronounced when there are small numbers of trials [20,21]. In addition, there are no established methods to account for crossovers from placebo to active therapy in indirect comparisons of oncology trials [16]. Perhaps due to these limitations, recent approaches to indirect comparisons and network meta-analyses have not been as widely used in oncology as in other therapeutic areas [16]. At the same time, the comparison of treatment outcomes across separate data sources, and appreciation of the inherent challenges and limitations of this approach, has a long history in oncology in the use of single arm trials with comparisons to historical controls.

To illustrate several challenges in the use of indirect comparisons in oncology, this paper develops comparative evidence for everolimus and sunitinib in the treatment of advanced pNET. Two trials are available in this indication: one comparing everolimus vs. placebo [22] and another comparing sunitinib vs. placebo [12]. Both trials allowed crossover from placebo to active therapy following disease progression. The present study indirectly compares OS and PFS between these trials, and addresses two common challenges that arise for indirect comparisons of new oncology treatments: 1) how to adjust for differences in multiple baseline characteristics among a small numbers of trials? and 2) how to indirectly compare OS across trials when placebo-arm OS is contaminated by crossover? These challenges are addressed by utilizing individual patient data (IPD) from the everolimus trial along with published summary data from the sunitinib trial in a matching-adjusted indirect comparison [20]. We discuss the implications of our findings for pNET and the strengths and limitations of matching-adjusted indirect comparisons for oncology trials.

## Methods

### Study populations

Evidence for the efficacy of everolimus and sunitinib in advanced pNET is based largely on two pivotal trials. Everolimus administered at 10 mg/day was compared to placebo in the multi-center phase III RAD001 in Advanced Neuroendocrine Tumors, third trial (RADIANT-3), conducted among 410 patients with advanced disease and documented progression in the previous 12 months. Sunitinib at 37.5 mg/day was compared to placebo in the randomized phase III trial A6181111, which enrolled 171 patients with advanced, progressive pNET before the study was discontinued early following an unplanned efficacy evaluation by the data and safety monitoring committee. Both trials reported significantly prolonged PFS with active therapy compared to placebo, by 11.0 vs. 4.6 months for everolimus vs. placebo [22] and 11.4 vs. 5.5 months for sunitinib vs. placebo [12], though the effect of sunitinib in A6181111 did not reach formal statistical significance after accounting for unplanned data looks and early stopping [13,14].

In both trials, patients who progressed on the placebo arm were allowed to crossover to active treatment. Additionally, patients randomized to placebo in A6181111 were allowed to crossover to sunitinib after the trial was stopped early. Individual patient data were obtained from Novartis Pharmaceuticals Corporation for RADIANT-3 up to the February 23, 2011 data cut-off. Published, aggregate results from A6181111 were obtained from the published literature [12] (data cutoff: April 15, 2009) and publically available regulatory briefing documents and reports [13,14] (data cutoff: June 1, 2010).

### Outcome measures

PFS, the primary endpoint in both trials, was defined as the time from randomization to the first investigator assessed documentation of disease progression according to Response Evaluation Criteria in Solid Tumors (RECIST) version 1.0 or death from any cause. In both trials, imaging was performed when progression was suspected or at scheduled assessments (every 12 weeks in RADIANT-3; week 5, 9 and every 8 weeks thereafter in A6181111). PFS was studied up to the data cutoff for the primary efficacy assessment in each trial: February 28, 2010 for RADIANT-3 and April 15, 2009 for A6181111 [12]. OS was available from both trials, and was studied up to the final February 23, 2011 data cutoff in RADIANT-3 and up to the last reported June 1, 2010 data cutoff for A6181111 [13]. OS and PFS outcomes were extracted from published data for A6181111 using digital figure estimation and verification vs. reported hazard ratios (HRs) [23-25]. Adverse event data were also considered from both studies, utilizing individual patient data from RADIANT-3 and rates of adverse events reported for A6181111 [12,26].

### Statistical methods

#### Adjusting for cross-trial differences in multiple baseline characteristics to compare PFS between trials

Indirect comparisons must account for cross-trial differences in patient populations. When indirect comparisons are based on published summary data, relative effect measures are used to account for cross-trial differences. For example, the hazard ratio (HR) for the effect of everolimus vs. placebo on PFS in RADIANT-3 could be compared to the HR for the effect of sunitinib vs. placebo in A6181111 [18-20]. An assumption underlying this comparison is that by measuring treatment effects relative to a common comparator arm, in this case placebo, the HRs account for any differences between the trial populations that could impact efficacy. This assumption may be difficult to accept if there are cross-trial differences in inclusion/exclusion criteria, baseline characteristics or study protocols that could impact the HRs [18]. With aggregate data for only two trials, it is not possible to use methods such as meta-regression to adjust for such differences [18]. By utilizing IPD for RADIANT-3 in the present study, it is possible to adjust for cross-trial differences in inclusion/exclusion criteria and observed baseline characteristics using a matching-adjusted indirect comparison [20,21,27].

Inclusion and exclusion criteria for RADIANT-3 and A6181111 are summarized in Additional file 1: Table S1. Both trials enrolled patients with advanced metastatic or unresectable pNETs that were well-or moderately-differentiated. However RADIANT-3 enrolled patients with a baseline World Health Organization (WHO) performance status of 2, whereas such patients were excluded in the design of A6181111 (A6181111 used the Eastern Cooperative Oncology Group [ECOG] assessment of performance status; this assessment is identical to the WHO assessment). To match eligibility criteria across the two trials, individual patients in RADIANT-3 with baseline WHO/ECOG performance status equal to 2 (ambulatory and capable of all self-care but unable to carry out any work activities; spending <50% of the day in bed) were removed from the analyses. Baseline characteristics were then compared between the selected RADIANT-3 population and the published A6181111 trial population using t-tests for continuous variables and chi-square tests for categorical variables.

A matching-adjusted indirect comparison was then used to adjust for cross-trial differences in observed baseline characteristics between RADIANT-3 and A6181111 [20]. This approach is similar to propensity score weighting [20,28]. Individual patients in RADIANT-3 were assigned weights such that, after weighting, the baseline medians of continuous variables and the baseline proportions for binary variables exactly matched those reported for A6181111. Each patient’s weight corresponded to his or her relative propensity for enrolling in RADIANT-3 versus A6181111. These relative propensities were estimated using a logistic regression model for trial enrolment that included all matched-on baseline characteristics as covariates [20]. Matched-on baseline characteristics consisted of all baseline variables that were available and consistently defined in both trials: age, sex, performance status (WHO/ECOG score of 0 versus 1), time since diagnosis (≥3 years versus <3 years), number of disease sites (1, 2, or ≥3), presence of distant metastases, prior use of somatostatin analogues, and prior systemic chemotherapy.

After matching baseline medians and proportions for these characteristics, PFS was compared for everolimus vs. sunitinib as the ratio of the within-trial HRs comparing each drug to placebo. In this analysis, the HR for PFS with everolimus versus placebo was estimated using a weighted Cox proportional hazards model fit to RADIANT-3, and was then compared to the published HR for sunitinib vs. placebo using the method of Bucher et al. in the matched samples [17]. For comparative purposes, the same analysis was performed using unweighted data from RADIANT-3.

#### Indirect comparison of OS when placebo-arm outcomes are contaminated by crossovers to active therapies

Crossovers from placebo to active therapy following disease progression are often permitted in oncology trials for ethical reasons. These crossovers do not impact assessments of PFS, but they can obscure potential drug effects on OS [29], and can complicate indirect comparisons of OS [16]. Indirect comparisons that rely on relative effect measures, such as HRs, will be invalid because placebo-arm OS outcomes do not provide a common comparator (since the crossed-to therapies differ between trials). The propensity to crossover following progression may also differ between trials due to differences in patient characteristics or study conduct.

In the present study, a matching-adjusted indirect comparison was used to compare OS between everolimus and sunitinib arms. The placebo arm data were not used due to crossovers in both trials. Individual patients from the everolimus arm in RADIANT-3 were included, and were assigned the same weights previously used to match baseline medians and proportions to A6181111. Figure-estimated OS data were utilized from the sunitinib arm in A6181111. These data were then analyzed using a weighted Cox proportional hazards model and weighted Kaplan-Meier estimates to compare OS between everolimus and sunitinib. The weighted analysis incorporated the same set of weights used to balance observed baseline characteristics, and in this way provided adjustment for observed cross-trial differences in these characteristics.

Although crossovers prevented the use of placebo-arm OS for this indirect comparison, placebo-arm PFS provided relevant information. In particular, placebo-arm PFS, which was not impacted by crossover, was compared across trials to assess the degree to which cross-trial differences in unobserved baseline characteristics may have confounded the indirect OS comparison. In this way, the comparison of placebo-arm PFS provided a negative control. If the trial populations were identical after weighting, no cross-trial differences in placebo-arm PFS would be expected. Any observed cross-trial differences in placebo-arm PFS, represented by a HR different from 1, would indicate the magnitude and direction of residual imbalance impacting PFS. This comparison was based on a weighted Cox proportional hazards model, incorporating weighted placebo-arm PFS data from RADIANT-3 and the figure-estimated PFS data from A6181111.

#### Comparing OS with everolimus versus the placebo arm in the sunitinib trial

Crossovers in RADIANT-3 and A6181111 complicate the assessment of drug effects on OS within each trial. Despite significantly prolonged PFS with everolimus vs. placebo in RADIANT-3, no significant difference in OS was detected between arms. Seventy three percent of patients randomized to placebo crossed over [22]. In A6181111, sunitinib was initially associated with prolonged OS versus placebo, at the time of early stopping based on an unplanned data look [12,13]. However, as patients were followed beyond early stopping, 69% of those randomized to placebo ultimately crossed over to sunitinib and no significant difference in OS was observed between sunitinib and placebo [13].

Though prolongation of PFS is a significant improvement in the treatment of advanced pNET, the lack of evidence for prolongation of OS with everolimus or sunitinib limits understanding of the full value of these treatments. Furthermore, the apparent early difference in OS between sunitinib and placebo, prior to early stopping of A6181111, raises the question of why a similar OS differences was not observed for everolimus in RADIANT-3. Possible explanations include cross-trial differences in patient populations, cross-trial differences in study conduct and differences in drug effects.

To address these possibilities, a matching-adjusted indirect comparison, based on a weighted Cox proportional hazards model and weighted Kaplan-Meier estimates, was used to compare OS between the everolimus arm in RADIANT-3 and the placebo arm in A6181111. Thus, the placebo arm in A6181111 was treated as an external control population. This analysis, and all other comparative analyses of PFS and OS performed in this study, and the data included in each, are summarized in Additional file 1: Table S2.

To summarize the effect of everolimus on OS after 1 year and after 2 years, the numbers needed to treat (NNT) were computed as the reciprocal of the estimated 1-year and 2-year OS differences between everolimus and the placebo arm in A6181111.

A 1-year NNT equal to 10, for example, would indicate that one death would be prevented in the first year of treatment for every 10 patients initiated with everolimus rather than placebo (with access to sunitinib after progression).

#### Comparison of adverse event rates

Rates of adverse events affecting >5% of patients on any arm of either trial were compared between RADIANT-3 and A6181111. Because adverse events were monitored over a longer time horizon in RADIANT-3 than in A6181111, adverse event data from the treatment and placebo arms of RADIANT-3 were censored at the maximum follow-up times of the respective A6181111 arms. The placebo-adjusted odds ratio of each adverse event for everolimus versus sunitinib was calculated as the odds ratio for everolimus versus placebo in the weighted RADIANT-3 population divided by the odds ratio for sunitinib versus placebo in A6181111 [17,20].

Analyses were conducted using SAS software version 9.2 (SAS Institute Inc., Cary, NC, USA). Statistical significance was assessed at the 5% level.

## Results

The intention-to-treat population in RADIANT-3 included 410 patients assigned to everolimus (N = 207) or placebo (N = 203). After excluding 15 patients with WHO scores of 2 at baseline, and 1 patient with missing baseline values, the RADIANT-3 study sample included 394 patients. The reported A6181111 trial population consisted of 171 patients randomized to sunitinib (N = 86) or placebo (N = 85) [12].

Prior to matching, and compared to baseline in A6181111, patients in RADIANT-3 were more likely to have a WHO/ECOG performance status of 0 (68.8% vs. 55.0%) and more likely to have previously used somatostatin analogues (49.2% vs. 36.3%), but less likely to have previously used systemic chemotherapy (48.7% vs. 69.0%). After matching adjustment, the medians and proportions of these and all other baseline characteristics available from both trials were exactly balanced (Table 1). In the matching-adjusted indirect comparison, with placebo arms serving as a common comparator, everolimus was associated with similar PFS compared to sunitinib (HR = 0.84, 95% CI = 0.46–1.53, p = 0.578; Table 2). Pre-matching analyses, based on the unweighted RADIANT-3 sample, produced similar results (Table 2).

**Table 1:** Baseline characteristics pre- and post-matching

**Baseline characteristics**^**a**^	**RADIANT-3 study sample**		**A6181111**
	**Pre-match**	**Post-match**	**As reported**
Median age (years)^b^	58.0	56.5	56.5
Age > 64 years	27.4	26.3	26.3
Female	44.9	52.0	52.0
WHO or ECOG^c^ performance status of 0	68.8^d^	55.0	55.0
Time since diagnosis ≥3 years	46.7	48.0	48.0
Number of disease sites			
1	28.2	31.4	31.4
2	36.8	33.7	33.7
≥3	35.0	34.9	34.9
Presence of distant metastases	96.2	94.7	94.7
Previous somatostatin analogues	49.2^d^	36.3	36.3
Previous systemic chemotherapy	48.7^d^	69.0	69.0

Notes:

^a^Reported as percentages, unless otherwise noted.

^b^For A6181111, 56.5 is the midpoint between reported medians on the active (56) and placebo (57) arms.

^c^An ECOG performance status of 0 was equated to a WHO performance status of 0.

^d^*P* < 0.05 for comparison vs. A6181111.

**Table 2:** Comparisons of progression-free and overall survival

**Comparison**	**Before matching**			**After matching**		
	**HR**	**95% CI**	**P-Value**	**HR**	**95% CI**	**P-Value**
*Progression-free survival*						
Everolimus vs. Placebo	0.38	0.29-0.49	<0.001	0.35	0.24-0.52	<0.001
Sunitinib vs. Placebo	0.42	0.26-0.66	<0.001	0.42	0.26-0.66	<0.001
Everolimus vs. Sunitinib^a^	0.90	0.53-1.53	0.695	0.84	0.46-1.53	0.578
*Overall survival*						
Everolimus vs. Placebo in A6181111	0.53	0.35-0.78	0.002	0.61	0.38-0.98	0.042
Everolimus vs. Sunitinib	0.69	0.46-1.05	0.087	0.81	0.49-1.31	0.383

Notes:

^a^Comparison based on the HR for everolimus vs. placebo divided by the HR for sunitinib vs. placebo.

In the matching-adjusted indirect comparison of OS, excluding placebo arm data due to crossovers, everolimus was associated with similar OS compared to sunitinib (HR = 0.81, 95% CI = 0.49–1.31, p = 0.383; Table 2). Placebo arm PFS was compared between the matched trial populations as a negative control. The HR for placebo-arm PFS in the weighted RADIANT-3 data versus A6181111 was 1.18 and did not differ significantly from 1 (95% confidence interval [CI] = 0.82-1.70; p = 0.363).

**Figure 1:**
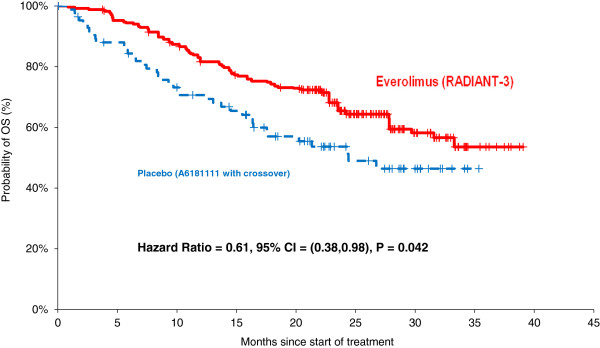
OS with Everolimus vs. placebo in A6181111 after matching.

In the matched populations, treatment with everolimus was associated with significantly longer OS compared to the placebo arm in A6181111, which included crossover to sunitinib after progression, (HR = 0.61, 95% CI = 0.38-0.98, p = 0.042, Table 2, Figure 1). Median OS with placebo in A6181111 was approximately 25 months. Median OS was not reached after 39 months of follow-up, the longest available, for patients randomized to everolimus in RADIANT-3. At one year of follow-up, the NNT with everolimus versus placebo in A6181111 to prevent one death was 8.3 (1-year OS was 82% with everolimus versus 70% with placebo in A6181111); at 2-years the NNT was 9.1 (2-year OS was 65% with everolimus versus 54% with placebo in A6181111).

Within the balanced trial populations, everolimus was associated with significantly higher placebo-adjusted rates of peripheral edema (odds ratio [OR] = 4.24; p = 0.011) and fever (OR = 3.22; p = 0.049) compared to sunitinib. Nearly all of these events were of grade 1 or 2 in severity in the everolimus arm, and occurrences of peripheral edema and fever of grade 3 or 4 did not differ significantly between everolimus and sunitinib. Conversely, placebo-adjusted rates of neutropenia (OR = 0.15; p = 0.049) and hypertension (OR = 0.19; p = 0.021) were significantly lower with everolimus than sunitinib, and more than one-third of neutropenia and hypertension events in the sunitinib arm were of grade 3 or 4. It should be noted that no adjustment was made for multiple comparisons in this exploratory analysis. No other adverse events showed statistically significant differences at the 5% level (see Additional file 1: Table S3 for complete results).

## Discussion

In the absence of a head-to-head randomized trial of everolimus and sunitinib, this study indirectly compared overall survival and progression-free survival with everolimus, sunitinib and the placebo arm in trial A6181111. By utilizing IPD from the trial of everolimus, a matching-adjusted indirect comparison was used to address methodological challenges that are common for new oncology treatments: adjusting for differences in multiple baseline characteristics among a small number of trials and comparing OS in the presence of crossovers crossovers. After adjusting for baseline differences between these trials, everolimus was associated with significantly prolonged OS compared to placebo in A6181111, which allowed crossover to sunitinib after progression. Everolimus was associated with similar PFS and OS compared to sunitinib.

The estimated effect on OS of everolimus versus placebo in A6181111 indicated that for every 10 advanced pNET patients treated with everolimus rather than placebo in A6181111, at least one additional patient is expected to live for two years. This estimated effect of everolimus on OS compared to an external control group corresponds to a clinically significant improvement in the treatment of advanced pNET. However, it is likely to be an underestimate of the effect of everolimus on OS relative to a pure placebo, since the placebo arm in A6181111 allowed crossover to sunitinib after progression or early stopping of the trial, which may have improved OS.

The estimated prolongation of OS with everolimus versus placebo in A6181111 differs from the absence of an effect on OS versus placebo in RADIANT-3 [6]. Prior to the present study, multiple explanations for differences in effects on OS between RADIANT-3 and A6181111 could have been considered, including cross-trial differences in patient populations, differences in study designs and differences in drug effects. The present study adjusted for observed baseline differences and assessed the likely direction of unobserved confounding. Since PFS was numerically longer on the placebo arm in A6181111 versus the placebo arm in RADIANT-3 after matching, it is more plausible that unobserved confounding favored longer OS in A6181111 versus RADIANT-3 than vice versa. (To argue otherwise would require factors that prolong PFS yet shorten OS.) Given these findings, the transient OS difference between sunitinib and placebo at early stopping of A6181111, and lack of OS difference between everolimus and placebo in RADIANT-3, should not be considered as greater evidence for an OS benefit with sunitinib than with everolimus. Rather, it seems that placebo-arm OS in A6181111 was unexpectedly short, despite numerically longer PFS compared to the placebo arm in RADIANT-3. Though the explanation for this could not be determined in the present study, it is notable that the aggregate extent of crossover from placebo was slightly greater in RADIANT-3 than in A6181111 (73% vs. 69%). Detailed information on the timing of crossover was not available from published data for A6181111. It was also not possible to compare patient characteristics at the time of crossover in these two trials.

Matching-adjusted indirect comparisons in the present study showed similar OS and PFS with everolimus compared to sunitinib. It should be noted that power to detect differences in PFS and OS between these two active therapies in a cross-trial comparison is limited, since each trial was powered to compare PFS versus placebo [26]. However, early stopping of A6181111, which was based on a trend towards longer PFS and OS with sunitinib versus placebo in an unplanned data look, may have led to overestimation of the true effect of sunitinib vs. placebo [13,14]. This potential overestimation in A6181111 may have led to underestimation of the effects of everolimus versus sunitinib on OS and PFS in the present study.

The present study also compared a large number of adverse event rates between everolimus and sunitinib. These analyses were not adjusted for multiple comparisons, and should be interpreted as exploratory. Many adverse events could not be compared because rates were not reported for A6181111, since they did not affect > 5% of the sunitinib arm (e.g., hyperglycemia, infections, pneumonia, and pruritis). It should be noted that the RADIANT-3 and A6181111 trials were powered to test within-trial differences in PFS, and the present study is likely to be underpowered to detect cross-trial differences in adverse event risk.

An indirect comparison of RADIANT-3 and A6181111 without adjustment for baseline differences, based only on comparing HRs across trials, would have been subject to confounding by observed baseline differences between trials. Before matching, notable differences were observed in performance status and prior treatments. These and other baseline differences could have impacted PFS and OS outcomes, even when measured as HRs. For example, HRs for the effect of everolimus versus placebo on PFS ranged from 0.21 to 0.47 across patient subgroups reported for RADIANT-3 [22] and HRs for PFS with sunitinib versus placebo ranged from 0.22 to 0.75 across subgroups reported for A6181111 [12]. Since baseline characteristics modify HRs, they could confound a cross-trial comparison of HRs. It should be noted that statistical significance of HR modification or of baseline differences is not necessary for significant confounding to occur. By balancing observed baseline characteristics across trials, the matching adjustment applied in the present study reduces the potential for observed characteristics to bias the cross trial comparison of outcomes, even if they do modify HRs relative to placebo.

Matching adjustment was also used in the present study to compare OS outcomes between active therapies, and between everolimus and the placebo arm in the sunitinib trial. In these comparisons, relative effect measures such as the HR could not be used due to crossovers on the placebo arms of both trials. However, it was possible to compare outcomes between trial populations that were balanced for all observed baseline characteristics, and to test the balance by comparing placebo arm PFS between trials. Matching-adjusted indirect comparisons versus external trial data can be viewed as an adjusted approach to comparisons against historical controls, which have a long history in oncology [30].

This study has several limitations. Most importantly, although this study used data from randomized controlled trials, the cross-trial comparisons are akin to observational studies, and have similar limitations [20]. In particular, as in any observational study, differences in unobserved patient characteristics or other systematic differences between trials could confound cross-trial comparisons of outcomes, despite matching on the observed characteristics. A related limitation is that the present study could only adjust for baseline characteristics that were available for both trials. There were also post-randomization differences in scheduled imaging assessments to detect disease progression. The impact of these differences is limited because comparisons were based on hazard ratios relative to placebo. The active and placebo arms shared the same imaging protocol within each trial. Comparisons of OS were not directly affected by differences in imaging schedules. After adjusting for available characteristics, residual imbalance in placebo-arm PFS, though not statistically significant, suggested prolonged PFS for placebo A6181111 versus placebo in RADIANT-3. A large head-to-head randomized trial of everolimus versus sunitinib would be needed to compare outcomes without the potential for unobserved confounding. If a randomized study were conducted, it would be interesting to compare the results with those of the present indirect comparison.

While head-to-head randomized trials provide the gold standard for comparative evidence, they are not always available for clinically relevant treatment comparisons, especially those involving new treatments. In the absence of a head-to-head trial, indirect comparisons based on individual patient data for all trials would provide the best comparative evidence. However, individual patient data are rarely available, to the same researchers, from pivotal trials of new oncology treatments developed by different manufactures. At the same time, indirect comparisons based only on aggregate data can face significant limitations when there are small numbers of trials and crossover is allowed, as is often the case for new oncology treatments. The present study has illustrated how these limitations can be addressed by combining individual patient data from trials of one treatment with published aggregate data from another treatment. Many oncology researchers engaged in comparative effectiveness research can access individual patient data for certain trials, but these data have not been widely used for indirect comparisons. Greater use of these individual patient data to adjust for cross-trial differences in indirect comparisons could increase the timeliness and reliability of comparative evidence for new oncology treatments.

## Conclusion

After adjusting for baseline differences between trials, treatment with everolimus was associated with a significantly lower hazard of death compared to placebo with access to sunitinib after disease progression. PFS and OS were similar with everolimus versus sunitinib. This indirect comparison could provide decision makers with informative comparative evidence, but may underestimate the efficacy of everolimus compared to sunitinib due to early stopping of the sunitinib trial and residual bias towards longer PFS in the sunitinib trial.

## Abbreviations

pNETs: Pancreatic neuroendocrine tumors; OS: Overall survival; IPD: Individual patient data; HRs: Hazard ratios; HR: Hazard ratio; WHO: World Health Organization; ECOG: Eastern Cooperative Oncology Group; NNT: Numbers needed to treat.

## Competing interests

This research was funded by Novartis Pharmaceuticals Corporation. JES, ES and EK are employees of Analysis Group Inc., which received funding from Novartis for this research. XW, JK and TH are employees of Novartis. BD and PM did not receive funding for this research.

## Authors’ contributions

JES, ES and EK designed the study, conducted analyses, interpreted results and drafted the manuscript. XW, JK and TH designed the study, interpreted results and reviewed manuscript drafts. BD and PM reviewed the study design, interpreted results and were involved in manuscript development. All authors read and approved the final manuscript.

## Supplementary Material

Additional file 1: Table S1 Comparison of Inclusion/Exclusion Criteria. **Table S2**: Comparative Analyses and Included Trial Arms. **Table S3**: Comparison of Adverse Event Rates. Click here for file
