# Supplementary material for: Everolimus and sunitinib for advanced pancreatic neuroendocrine tumors: a matching-adjusted indirect comparison
Source: Exp Hematol Oncol. 2013 Dec 6;2:32. doi: 10.1186/2162-3619-2-32 (PMC4175512; doi:10.1186/2162-3619-2-32)
Supplement: Additional file 1: Table S1 — Comparison of Inclusion/Exclusion Criteria. Table S2: Comparative Analyses and Included Trial Arms. Table S3: Comparison of Adverse Event Rates. [file 2162-3619-2-32-S1.docx]

**Supplemental Table 1: Comparison of Inclusion/Exclusion Criteria**

| **Criteria** | **RADIANT-3** | **A6181111** |
| --- | --- | --- |
| Tumor status | Advanced | Advanced |
| Differentiation | Well- or moderately-differentiated | Well- or moderately-differentiated^a^ |
| Progression | Documented within  the previous 12 months | Documented within  the previous 12 months |
| Measurable disease | Present | Present |
| Performance status^b^ | WHO score of 0, 1 or 2 | ECOG score of 0 or 1 |

**Notes:**

1. Well-differentiated tumors include moderately differentiated tumors in A6181111.
2. An ECOG performance status of 0 or 1 was equated to a WHO performance status of 0 or 1, respectively.

**Supplemental Table 2.  Comparative Analyses and Included Trial Arms**

| **Analysis** | **RADIANT-3 Study Sample (Post-Match)** | |  | **A6181111** | |
| --- | --- | --- | --- | --- | --- |
|  | **Everolimus** | **Placebo** |  | **Sunitinib** | **Placebo** |
| Comparison of PFS between everolimus and sunitinib | X | X |  | X | X |
| Comparison of OS between everolimus and sunitinib | X |  |  | X |  |
| Comparison of OS between everolimus and placebo in A6181111 | X |  |  |  | X |
| Assessment of balance after matching |  | X |  |  | X |

**Supplemental Table 3: Comparison of Adverse Event Rates**

| **Outcomes** | **RADIANT-3 Post-Match** | |  | **A6181111** | |  | **Everolimus vs.  Sunitinib** | |
| --- | --- | --- | --- | --- | --- | --- | --- | --- |
|  | **Everolimus** | **Placebo** |  | **Sunitinib** | **Placebo** |  | **Odds Ratio** | **P-value** |
| Diarrhea | 45.9% | 25.0% |  | 59.0% | 39.0% |  | 1.1295 | 0.7695 |
| Grade 1 or 2 | 39.6% | 23.3% |  | 54.2% | 36.6% |  | 1.0499 | 0.9078 |
| Grade 3 or 4 | 6.3% | 1.7% |  | 4.8% | 2.4% |  | 1.9268 | 0.5405 |
| Nausea | 31.8% | 32.1% |  | 44.6% | 29.3% |  | 0.5084 | 0.1056 |
| Grade 1 or 2 | 30.4% | 29.7% |  | 43.4% | 28.0% |  | 0.5246 | 0.1272 |
| Grade 3 or 4 | 1.5% | 2.4% |  | 1.2% | 1.2% |  | 0.6221 | 0.7709 |
| Asthenia | 18.7% | 21.2% |  | 33.7% | 26.8% |  | 0.6128 | 0.2933 |
| Grade 1 or 2 | 15.7% | 18.2% |  | 28.9% | 23.2% |  | 0.6216 | 0.3355 |
| Grade 3 or 4 | 2.9% | 3.0% |  | 4.8% | 3.7% |  | 0.7253 | 0.7605 |
| Vomiting | 28.2% | 26.2% |  | 33.7% | 30.5% |  | 0.9576 | 0.9212 |
| Grade 1 or 2 | 27.4% | 22.3% |  | 33.7% | 28.0% |  | 1.0072 | 0.9872 |
| Grade 3 or 4 | 0.9% | 3.9% |  | 0.0% | 2.4% |  | 0.9386 | 0.9667 |
| Fatigue | 41.2% | 25.5% |  | 32.5% | 26.8% |  | 1.5615 | 0.3038 |
| Grade 1 or 2 | 38.5% | 23.0% |  | 27.7% | 18.3% |  | 1.2239 | 0.6641 |
| Grade 3 or 4 | 2.7% | 2.5% |  | 4.8% | 8.5% |  | 2.0494 | 0.4575 |
| Neutropenia | 6.0% | 3.7% |  | 28.9% | 3.7% |  | 0.1543 | 0.0491 |
| Grade 1 or 2 | 2.2% | 0.0% |  | 16.9% | 3.7% |  | 1.2306 | 0.8629 |
| Grade 3 or 4 | 3.9% | 3.7% |  | 12.0% | 0.0% |  | 0.0826 | 0.0495 |
| Abdominal pain | 18.8% | 27.1% |  | 27.7% | 31.7% |  | 0.7547 | 0.5287 |
| Grade 1 or 2 | 15.7% | 20.1% |  | 22.9% | 22.0% |  | 0.7003 | 0.4621 |
| Grade 3 or 4 | 3.1% | 6.9% |  | 4.8% | 9.8% |  | 0.9085 | 0.9156 |
| Hypertension | 8.5% | 6.6% |  | 26.5% | 4.9% |  | 0.1858 | 0.0205 |
| Grade 1 or 2 | 7.9% | 6.3% |  | 16.9% | 3.7% |  | 0.2394 | 0.0768 |
| Grade 3 or 4 | 0.6% | 0.4% |  | 9.6% | 1.2% |  | 0.1972 | 0.3277 |
| Palmar-plantar erythrodysesthesia syndrome | 3.0% | 0.0% |  | 22.9% | 2.4% |  | 0.8328 | 0.8871 |
| Grade 1 or 2 | 2.1% | 0.0% |  | 16.9% | 2.4% |  | 0.8770 | 0.9214 |
| Grade 3 or 4 | 0.9% | 0.0% |  | 6.0% | 0.0% |  | 0.4378 | 0.6067 |
| Stomatitis | 68.7% | 17.6% |  | 21.7% | 2.4% |  | 0.9238 | 0.9229 |
| Grade 1 or 2 | 61.5% | 17.6% |  | 18.1% | 2.4% |  | 0.8460 | 0.8390 |
| Grade 3 or 4 | 7.1% | 0.0% |  | 3.6% | 0.0% |  | 3.9049 | 0.3740 |
| Dysgeusia | 18.9% | 4.7% |  | 20.5% | 4.9% |  | 0.9369 | 0.9294 |
| Grade 1 or 2 | 18.9% | 4.7% |  | 20.5% | 4.9% |  | 0.9369 | 0.9294 |
| Grade 3 or 4 | 0.0% | 0.0% |  | 0.0% | 0.0% |  | 0.9842 | 0.9957 |
| Epistaxis | 22.3% | 0.9% |  | 20.5% | 4.9% |  | 5.9582 | 0.0849 |
| Grade 1 or 2 | 22.3% | 0.9% |  | 19.3% | 4.9% |  | 6.4265 | 0.0730 |
| Grade 3 or 4 | 0.0% | 0.0% |  | 1.2% | 0.0% |  | 0.4861 | 0.7035 |
| Headache | 30.4% | 16.2% |  | 18.1% | 13.4% |  | 1.5859 | 0.3858 |
| Grade 1 or 2 | 29.5% | 15.5% |  | 18.1% | 12.2% |  | 1.4336 | 0.5077 |
| Grade 3 or 4 | 0.9% | 0.7% |  | 0.0% | 1.2% |  | 2.4006 | 0.5722 |

**Supplemental Table 3: Comparison of Adverse Event Rates (continued)**

| **Outcomes** | **RADIANT-3 Post-Match** | |  | **A6181111** | |  | **Everolimus vs.  Sunitinib** | |
| --- | --- | --- | --- | --- | --- | --- | --- | --- |
|  | **Everolimus** | **Placebo** |  | **Sunitinib** | **Placebo** |  | **Odds Ratio** | **P-value** |
| Insomnia | 12.5% | 6.4% |  | 18.1% | 12.2% |  | 1.3119 | 0.6482 |
| Grade 1 or 2 | 12.5% | 6.4% |  | 18.1% | 12.2% |  | 1.3119 | 0.6482 |
| Grade 3 or 4 | 0.0% | 0.0% |  | 0.0% | 0.0% |  | 0.9842 | 0.9957 |
| Rash | 53.3% | 16.3% |  | 18.1% | 4.9% |  | 1.3589 | 0.6419 |
| Grade 1 or 2 | 52.9% | 16.3% |  | 18.1% | 4.9% |  | 1.3391 | 0.6579 |
| Grade 3 or 4 | 0.4% | 0.0% |  | 0.0% | 0.0% |  | 1.7093 | 0.7740 |
| Thrombocytopenia | 14.0% | 1.4% |  | 16.9% | 4.9% |  | 2.9220 | 0.2674 |
| Grade 1 or 2 | 9.2% | 1.4% |  | 13.3% | 4.9% |  | 2.4166 | 0.3693 |
| Grade 3 or 4 | 4.8% | 0.0% |  | 3.6% | 0.0% |  | 2.6412 | 0.5366 |
| Mucosal inflammation | 0.6% | 0.0% |  | 15.7% | 7.3% |  | 0.9635 | 0.9807 |
| Grade 1 or 2 | 0.0% | 0.0% |  | 14.5% | 7.3% |  | 0.4886 | 0.6364 |
| Grade 3 or 4 | 0.6% | 0.0% |  | 1.2% | 0.0% |  | 1.0472 | 0.9765 |
| Weight decreased | 29.0% | 11.1% |  | 15.7% | 11.0% |  | 2.1614 | 0.1879 |
| Grade 1 or 2 | 28.0% | 11.1% |  | 14.5% | 11.0% |  | 2.2635 | 0.1671 |
| Grade 3 or 4 | 1.0% | 0.0% |  | 1.2% | 0.0% |  | 1.4516 | 0.8299 |
| Constipation | 13.5% | 14.8% |  | 14.5% | 19.5% |  | 1.2921 | 0.6392 |
| Grade 1 or 2 | 13.5% | 14.8% |  | 14.5% | 18.3% |  | 1.1932 | 0.7481 |
| Grade 3 or 4 | 0.0% | 0.0% |  | 0.0% | 1.2% |  | 1.9929 | 0.7119 |
| Back pain | 11.1% | 12.0% |  | 12.0% | 17.1% |  | 1.3765 | 0.5851 |
| Grade 1 or 2 | 10.3% | 11.0% |  | 12.0% | 12.2% |  | 0.9475 | 0.9307 |
| Grade 3 or 4 | 0.8% | 1.0% |  | 0.0% | 4.9% |  | 4.3768 | 0.2912 |
| Upper abdominal pain | 16.3% | 9.7% |  | 13.3% | 7.3% |  | 0.9366 | 0.9225 |
| Grade 1 or 2 | 13.7% | 7.8% |  | 12.0% | 7.3% |  | 1.0735 | 0.9172 |
| Grade 3 or 4 | 2.7% | 1.9% |  | 1.2% | 0.0% |  | 0.6611 | 0.7992 |
| Anemia | 23.6% | 12.9% |  | 6.0% | 9.8% |  | 3.5310 | 0.0714 |
| Grade 1 or 2 | 16.5% | 11.6% |  | 4.8% | 8.5% |  | 2.7733 | 0.1856 |
| Grade 3 or 4 | 7.1% | 1.3% |  | 1.2% | 1.2% |  | 6.0046 | 0.2571 |
| Cough | 18.3% | 12.5% |  | 8.4% | 8.5% |  | 1.5894 | 0.4749 |
| Grade 1 or 2 | 18.3% | 12.5% |  | 8.4% | 8.5% |  | 1.5894 | 0.4749 |
| Grade 3 or 4 | 0.0% | 0.0% |  | 0.0% | 0.0% |  | 0.9842 | 0.9957 |
| Decreased appetite | 28.8% | 19.6% |  | 6.0% | 4.9% |  | 1.3278 | 0.7055 |
| Grade 1 or 2 | 27.1% | 18.9% |  | 6.0% | 4.9% |  | 1.2766 | 0.7456 |
| Grade 3 or 4 | 1.8% | 0.7% |  | 0.0% | 0.0% |  | 1.8264 | 0.7096 |
| Dyspnea | 14.2% | 6.9% |  | 12.0% | 14.6% |  | 2.7939 | 0.0932 |
| Grade 1 or 2 | 12.7% | 6.3% |  | 10.8% | 13.4% |  | 2.7368 | 0.1162 |
| Grade 3 or 4 | 1.6% | 0.6% |  | 1.2% | 1.2% |  | 2.6866 | 0.5870 |
| Edema peripheral | 34.2% | 13.3% |  | 12.0% | 14.6% |  | 4.2357 | 0.0110 |
| Grade 1 or 2 | 33.2% | 12.2% |  | 12.0% | 13.4% |  | 4.0575 | 0.0155 |
| Grade 3 or 4 | 1.0% | 1.1% |  | 0.0% | 1.2% |  | 1.8281 | 0.7000 |
| Pyrexia | 30.3% | 12.0% |  | 10.8% | 11.0% |  | 3.2234 | 0.0490 |
| Grade 1 or 2 | 29.5% | 10.8% |  | 9.6% | 11.0% |  | 4.0011 | 0.0225 |
| Grade 3 or 4 | 0.8% | 1.3% |  | 1.2% | 0.0% |  | 0.3722 | 0.5314 |
